# Evaluating personalized circulating tumor DNA detection for early‐stage lung cancer

**DOI:** 10.1002/cam4.6817

**Published:** 2023-12-19

**Authors:** Haihua Huang, Zhentian Kai, Yuchen Wang, Xiaomiao Zhang, Jin Wang, Wei Zhang, Qian Xue, Hang Zhang, Hansong Jin, Peize Meng, Shuilong Zhang, Yueyue Yang, Honghua Yang, Wanning Liang, Guangbing Zha, Peng Luo, Yan Xu, Weiwei Shi, Zheng Ruan

**Affiliations:** ^1^ Department of Thoracic Surgery Shanghai First People's Hospital Shanghai China; ^2^ Department of research and Development, Zhejiang Shaoxing Topgen Biomedical Technology Co., Ltd. Shanghai China

**Keywords:** biomarkers, biotechnology, genomics, lung cancer, molecular diagnosis

## Abstract

Circulating tumor DNA (ctDNA) has been widely used as a minimally invasive biomarker in clinical routine. However, a number of factors such as panel design, sample quality, patients' disease stages are known to influence ctDNA detection sensitivity. In this study, we systematically evaluated common factors associated with the variability of ctDNA detection in plasma and investigated ctDNA abundance in bronchoalveolar lavage (BAL). Whole exome profiling was conducted on 61 tumor tissue samples to identify tumor‐specific variants, which were then used to design personalized assay MarRyDa® for ctDNA detection. DNA extracted from BAL fluid and plasma were genotyped using MarRyDa® platform. Our analysis showed that histological subtypes and disease stages had significant differences in ctDNA detection rate. Furthermore, we found that DNA purified from BAL supernatants contains the highest levels of ctDNA compared with BAL precipitates and plasma; therefore, utilizing BAL supernatants for tumor detection might provide additional benefits. Finally, we demonstrated that tumor cellularity played significant roles in the design of personalized ctDNA panel which eventually impacts ctDNA detection sensitivity. We suggest setting a flexible criteria for sample quality control and utilization of BAL might benefit more patients in clinics.

## 1 INTRODUCTION

Lung cancer is one of the most prevalent and lethal types of cancer, with non‐small cell lung cancer (NSCLC) being the predominant type, constituting approximately 85% of all lung cancer cases.^1^, ^2^ Despite advances in early diagnosis technology and personalized treatment therapy, lung malignancy remains as the leading cause of all cancer‐related deaths globally, accounting for approximately 20% of all such deaths.^2^ The high mortality rate of lung cancer is mainly attributed to recurrence after curative‐intent surgery. Stage I NSCLC patients have reported relapse rate ranging from 27% to 38%,^3^ while patients with stage II–III lung cancer have the such rate of up to 60%.^4^ The prognosis for patients with recurrent lung cancer is very poor, with 5‐year survival rates at only 4%, leaving limited treatment options and a high risk of mortality.^5^ Currently, radiographic imaging is the gold standard for clinical routine surveillance.^6^ Such surveillance technology, however, has limitations in both sensitivity and specificity. On the one hand, small tumors may be invisible especially if they are located in areas that are difficult to visualize. On the other hand, pseudo‐progression where tumor enlarges under radiographic scans initially after immunotherapy may cause unnecessary anxiety to patients.^7^ Therefore, there is an urgent need for a both sensitive and specific biomarker that could reveal the recurrence of tumor at early stages.

Liquid biopsy, a non‐invasive diagnostic technology, exploring on various biomarkers including exosomes, circulating tumor cells and cell‐free DNA (cfDNA) in blood and/or other body fluids.^8^ Compared with tissue biopsy, liquid biopsy can circumvent several hurdles such as temporal or spatial tumor heterogeneity in tumor tissue, traumatic invasion and a high risk of complication for conventional biopsy approaches.^9^ Liquid biopsy provides a better accessibility to specimens, potentially increasing screening uptake and allowing continuous monitor.^10^ cfDNA, a type of DNA released from cells via apoptosis and necrosis, is one of the most studied biomarkers in liquid biopsy. Currently, most studies on cfDNA focus on peripheral blood.^11^ Nevertheless, the detection sensitivity of cfDNA on peripheral blood could be suboptimal for cancer patients with low‐tumor burden, which largely impedes its capacity for tumor surveillance and profiling.^12^ An awareness that some other types of body fluids may have a higher concentration of circulating tumor DNA (ctDNA) than blood has veered the research direction from peripheral blood to biofluid‐based sampling.^13^ Researches on other types of body fluids such as urine for genito‐renal cancers,^14^ ascites for gynecologic cancers,^15^ and cerebrospinal fluid for gliomas^16^ have been proven to be enriched for tumor‐derived molecular markers. Similarly, bronchoalveolar lavage (BAL) fluid, a type of proximal fluid collected during bronchoscopy, served as a superior target for profiling lung cancer genomes, enriching in ctDNA than plasma.^17^

In recent years, a number of personalized ctDNA detection assays that utilized patient‐specific tumor mutation have been developed, aiming to improve ctDNA detection sensitivity.^18^, ^19^, ^20^ In contrast to panel‐based ctDNA detection assay or tumor‐naive/agnostic assay, personalized or tumor‐inform assay relies on genomic profiling of tumor tissue to identified patient‐specific tumor variants. However, improved detection sensitivity comes with increasing turnaround time and aggravating economic burden. Moreover, the high complexity nature of tumor‐informed assay also challenges quality control steps. Patients whose tissue samples failed in quality control step cannot have their personalized ctDNA assay designed, leaving themselves limits of surveillance approaches.

In this study, we investigated several common conditions in clinics that might lead to reducing ctDNA detection power in tumor‐informed assay and hypothesized that BALF from patients with lung cancer is more reliable for ctDNA detection in comparison to peripheral blood. To explore this question, we utilized whole exon sequencing (WES) to identify mutations in NSCLC tissue, and further designed personalized ctDNA detection assay MaRryDa® for BALF and peripheral blood ctDNA detection.

## 2 METHOD

### 2.1 Patients enrollment and sample collection

A total of 62 patients were prospectively enrolled at Shanghai General Hospital, Shanghai, China, from July 2022 to December 2022. The study was approved by Shanghai General Hospital Institutional Review Board (2023‐176). Tumor tissues were obtained through surgery or needle biopsy. Blood samples were collected (20 mL in EDTA tubes) before treatment and subjected to centrifugation within 1 h after collection. Both plasma and white blood cells (WBC) were conserved at −80°C. BAL were obtained during bronchoscopic procedures. Lavage was performed using 45–60 mL of saline in 1–2 fractions after wedging the bronchoscope. The aspirated lavage was returned to specimen bottle using manual suction and then placed into 1:100 V/v 0.5 M for cell stabilization.

### 2.2 Sample processing, library preparation, and sequencing

DNA extraction from tumor tissues, WBC and BAL precipitates were performed using DNeasy Blood & Tissue Kit (Qiagen), while BAL supernatants and plasma were extracted using QIAamp Circulating Nucleic Acid Kit (Qiagen). Tumor tissue and WBC sequencing libraries were generated using KAPA Hyper Prep Kit according to the manufacturer's instruction. Genomic DNA capture was performed on VAHTS Target Capture Core Exome Panel (Vazyme Biotech). Plasma libraries were prepared using Scale ssDNA‐seq Lib Prep Kit (ABclonal Science). At least 30 ng of cfDNA were required in library preparation. Samples were sequenced using 150PE on Illumina Novaseq 6000 at a mean coverage depth of 500× and 200× for tumor tissue and WBC, respectively.

### 2.3 Variant calling and prioritizing of somatic mutations

Raw reads were trimmed to remove adapter sequences and low‐quality sequences using Fastp^21^ and aligned to human reference genome hg19 using BWA‐MEM algorithm (accelerated version provided by commercial software Sentieon).^22^, ^23^ Base recalibration and duplicates flagging were also performed by Sentieon. Germline and somatic variants were identified by Sentieon DNAseq and TNseq, respectively. Next, all variants were subjected to a filter strategy to remove artifacts and potential clonal hematopoiesis. Variants with low allele frequency (VAF <5%) and/or low quality (not flagged as PASS) were removed from further analysis. To avoid clonal hematopoiesis, variants that occurred in both tumor and WBC, with VAF >1% in WBC were also removed. Sequenza was used for allele‐specific copy numberprofiling and tumor cellularity estimation.^24^ Cellularity was manually adjusted to avoid overestimation. Mean VAF of all somatic point variants was used as the reference during manual adjustment. To retain as many variants as possible, we prioritized somatic mutation instead of selecting a subset of variants. PyClone was used for clonality calculation.^25^ All variants were used as the reservoir for next step of primer design and ranked based on: (1) whether it is a driver mutation; (2) whether it is a clonal or subclonal mutation; (3) its VAF. Variants that are both driver and clonal and have the highest VAF will be ranked at the top. While passenger or subclonal mutation with low VAF will be ranked at the bottom.

### 2.4 Preparation of MarRyDa® library

Personalized ctDNA detection assay MarRyDa® was designed based on multiplex PCR amplification of at most 20 personalized tumor mutations, which were identified by WES of its corresponding tumor tissue and WBC. For each patient, a customized panel of 2–20 pairs of primers was created. 6 bp of random bases, acting as the unimolecular identifier (UMI), were incorporated into each end of the primer (UMI structure: NNWNNW) to facilitate error suppression during ctDNA analysis. The initial 6 cycles of PCR were used to enrich the DNA of interest. Another set of primers was designed to cover and amplify the adapter sequences of previous primers by performing subsequent 22 cycles of PCR. Primer design was conducted using MFEPrimer,^26^ with primer size set to 17–25 bp, product size set to 60–100 bp and primer temperature set to 58–62. For each sample, a maximum of 20 variants were evaluated for the multiplex primer design. If variants failed during primer designing, then backup variants (if available) will be used to fill the gap. The process will continue until 20 variants are designed or no backup variants are available. Samples with less than 2 variants being successfully designed for primer will be removed from the following analysis.

### 2.5 Quality control and ctDNA detection in MarRyDa®

Raw reads were trimmed with Fastp with high‐quality standards (at least 30% of bases have quality above Q30).^21^ UMIs were then extracted from both ends of reads and aligned to hg19 reference genome using Sentieon UMI pipeline.^27^ Reads belonging to the same UMI family group were merged into a consensus read with base quality adjusted. Quality control was performed after reads alignment. Samples with Q30 rate below 80%, or mean depth coverage below 100,000× was removed from further analysis. Sentieon TNscope was used for variant detection with a minimal allele fraction set to 0.02%. At least 8 high‐quality reads (≥Q40) were required to make a valid calling. Samples with at least two variants identified were deemed as ctDNA‐positive. ctDNA fraction was measured by the mean VAF across all designed loci (including 0.0%).

### 2.6 Statistical analysis

All statistical analysis was performed using R 4.0.0. Wilcoxon signed‐rank test was used to compare differences in ctDNA fractions between any two groups. Kendall's rank correlation test was used to analyze the relationship between mutation burden and tumor cellularity. Random sampling with replacement was performed on 20 loci of ctDNA‐positive patients to generate subsets of 20 loci. For each total loci number of subset panel, detection sensitivity was averaged over 1000 samplings of all ctDNA‐positive patients.

## 3 RESULTS

### 3.1 Patients characteristics and study workflow

Overall, 61 lung cancer patients were enrolled in our study between June 2022 and December 2022, including 30 males and 31 females with a median age of 65 years (range: 34–82). Based on histological classification, there were 34 lung adenocarcinomas (LUAD), 12 lung squamous cell carcinomas (LUSC), 9 minimal invasive carcinomas (MIA), and 6 other types. A strong association between gender and histological subtypes was observed, with LUSC occurring exclusively in male and MIA mostly occurs in female (7/9). Most patients were diagnosed at early stages of lung cancer (73.8%, stage I: 38; stage II: 7).

**FIGURE 1:**
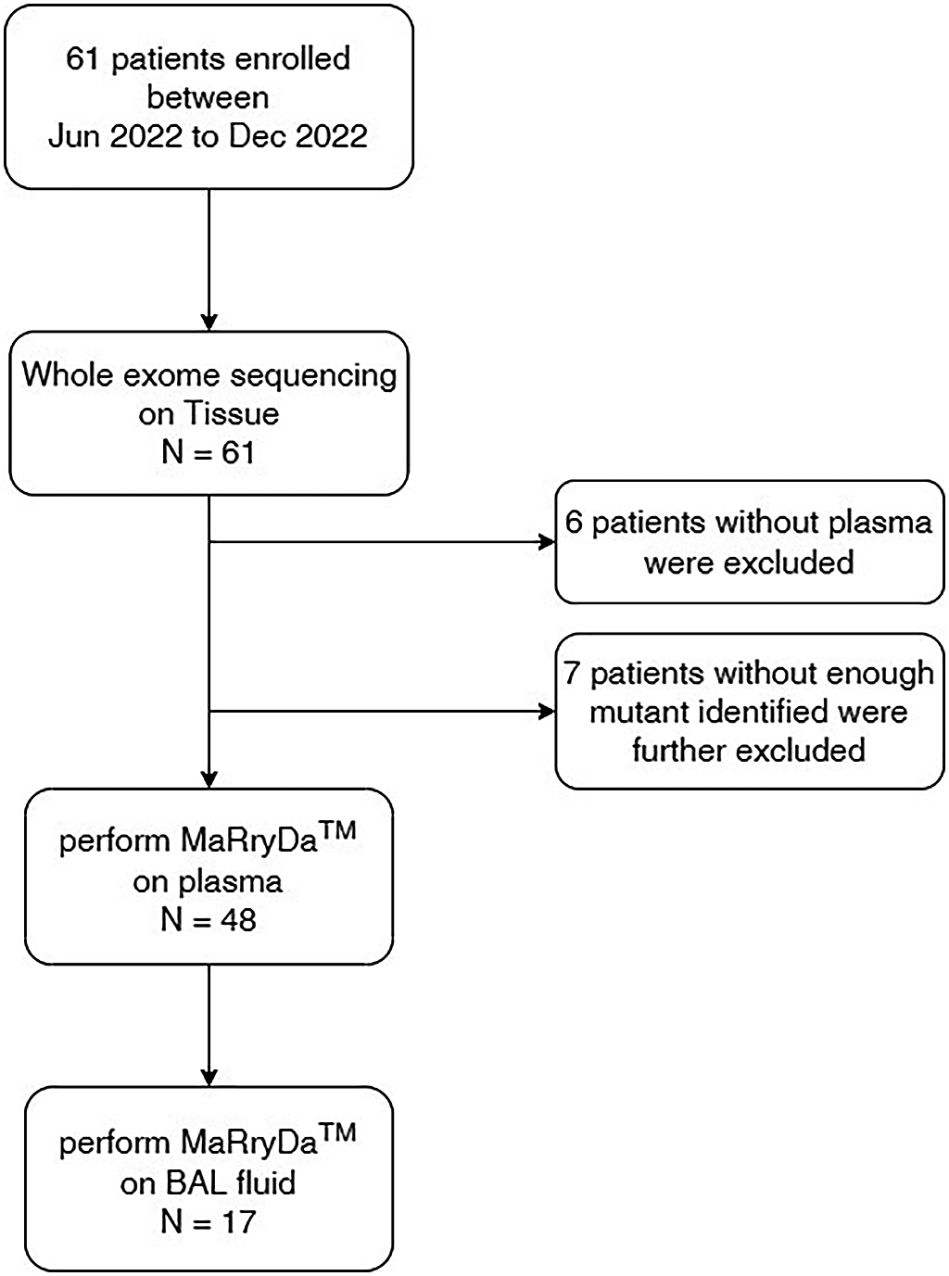
Study workflow: 61 patients with lung cancer were recruited and subjected to whole exome sequencing to identify patient‐specific somatic mutations. MaRryDa® were successfully designed for 48 patients with plasma available. For 17 patients with BAL fluid available, MaRryDa® were performed on both BAL supernatants and precipitates. BAL, bronchoalveolar lavage.

As illustrated in Figure 1, Whole exome sequencing was performed on all tissue samples of 61 lung cancer patients. Based on the result of WES data, personalized ctDNA detection panels MarRyDa® were designed on 55 patients with available pre‐treatment plasma. During the design, 7 patients with ≤2 mutants identified in WES were further excluded. Lastly, MarRyDa® was performed on both supernatants and precipitates of 17 patients with BAL fluid available.

### 3.2 Whole genome profiling on cohort

**FIGURE 2:**
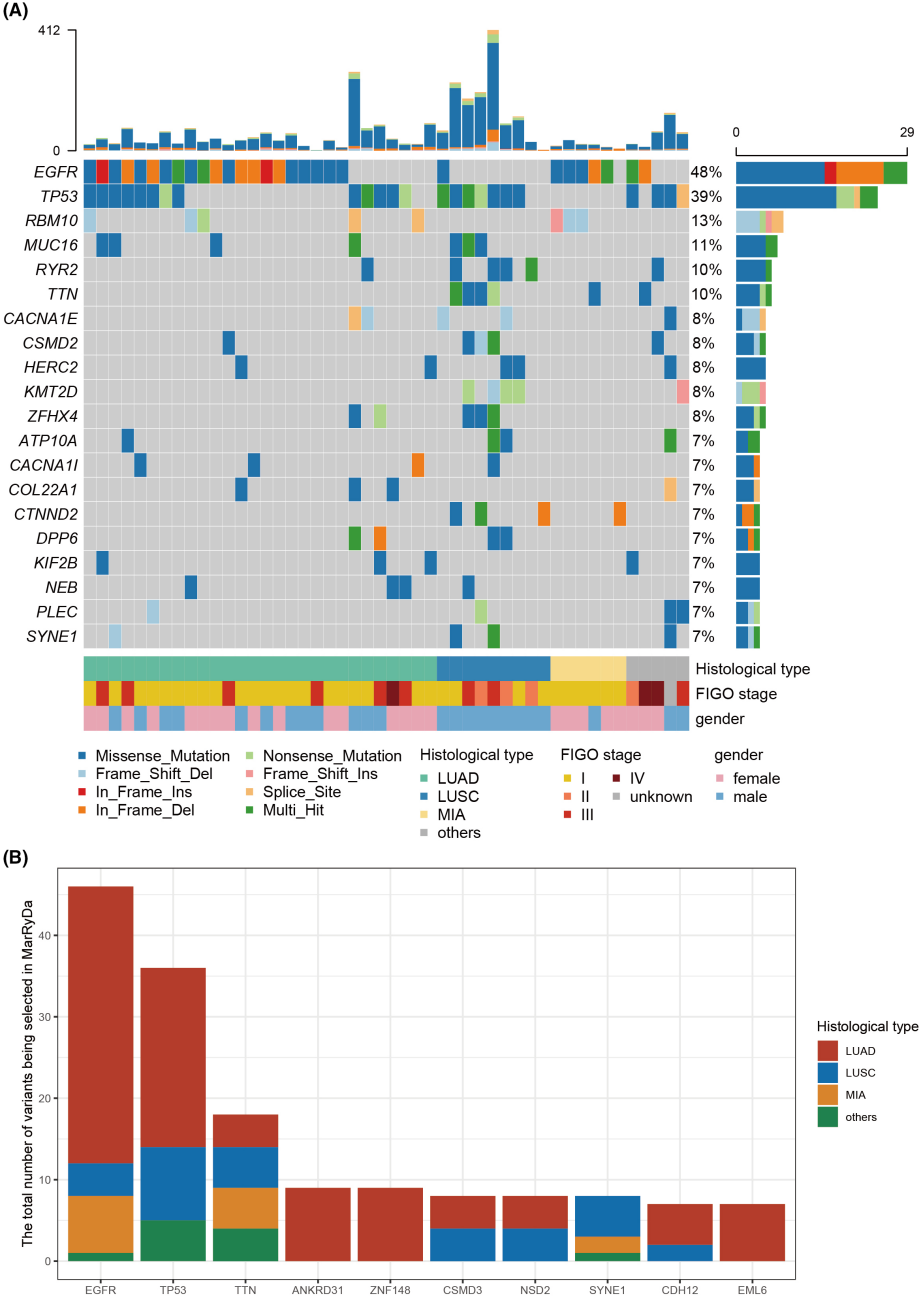
Mutations landscape: (A) Oncoplot of mutations in 61 lung cancer patients. Top 20 mutated genes are listed and ranked on the y‐axis. Patients are shown on the x‐axis and grouped based on histological types. The top panel shows the total number of mutants for each patients. The right panel showed the number of variants in different types of mutation for each genes. (B) Top 10 genes being selected in personalized ctDNA detection assay. ctDNA, circulating tumor DNA.

Whole exome sequencing totally identified 5471 mutations for the entire cohort (Figure 2A), with EGFR being the principally mutated gene (29/61, 47.5%), and TP53 being the second most altered gene (24/61, 39.3%) (Table S1). Among all clinical aspects, histological subtypes played the most important role in mutation spectrum. LUSC had the highest level of mutation burden (median: 137) whereas LUAD and MIA only had a median of 50 and 25 variants being detected. In adenocarcinomas cohort, EGFR was the primarily altered gene for both LUAD and MIA with a frequency of 62% and 56%, respectively. RBM10 ranked as the second and third most mutated gene in MIA and LUAD with a frequency of 33% and 15%, respectively. In squamous cell cohort, TP53 had the highest mutation rate (9/12, 58%) and KMT2D had the second highest mutation rate (4/12, 33%).

We subsequently investigated influences of mutational spectrum on mutant selection for personalized ctDNA assay (Figure 2B). Overall, 21/48 (43.7%) patients had all 20 locus being designed, while 6 patients had less than 10 locus. EGFR and TP53 remained as the top 2 genes selected; however, the rest of 8 top selected genes showed limited overlaps with top mutated genes. Interestingly, we found that SYNE1, a gene that only altered in 4 out of 61 patients, was almost exclusively being selected in LUSC. Contribution to SYNE1 gene mostly came from one particular patient with 8 SYNE1 mutants identified. Apart from top 3 selected genes, no significant difference in the number of times being selected was observed in the rest of the genes.

### 3.3 Analysis of ctDNA detection on pre‐treatment plasma

**FIGURE 3:**
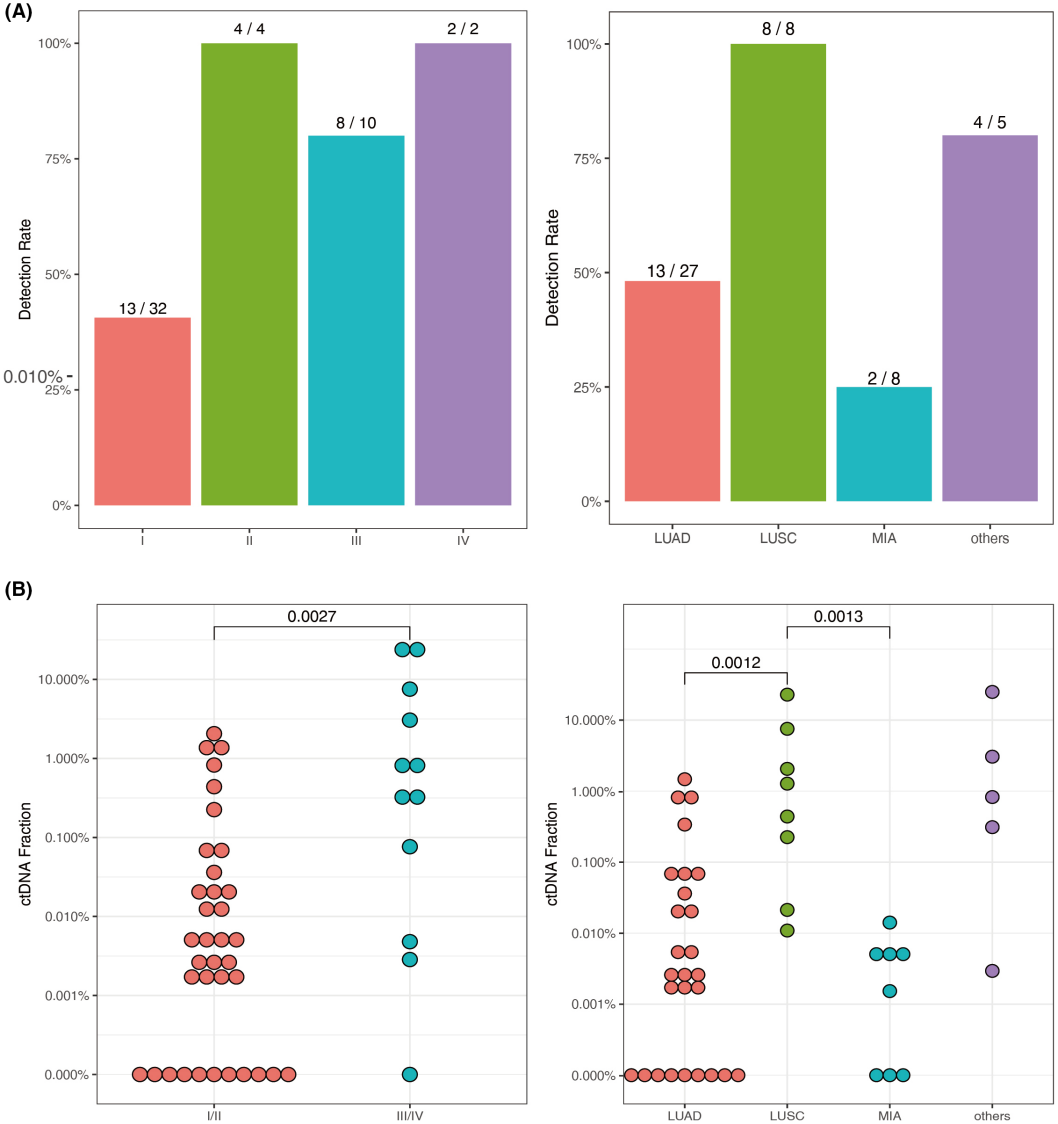
ctDNA detection on 48 patients' pre‐treatment plasma. (A) Bar chart of detection rate based on disease stages and histological subtypes. (B) Dot plot of ctDNA fractions based on disease stages and histological subtypes. ctDNA, circulating tumor DNA.

Next, we evaluated our platform performance on 48 pre‐treatment plasma samples. In total, ctDNA was detected in 27/48 (56.3%) of samples, showing a significant association with disease stage and histological subtype (Variants listed in Table S2). Only 13/32 (40.6%) of stage I cancer patients were detected in MarRyDa®, while most stage II/III/IV cancer patients can be detected (14/16, 87.5%, Figure 3A). All of LUSC patients had detectable ctDNA levels (8/8) in their plasma, compared with 48.1% (13/27) in LUAD patients. MIA, on the other hand, had the lowest detection rate of 25.0% (2/8) in our cohort. This observation was consistent with level of ctDNA fractions. LUSC had a significantly higher ctDNA fraction than LUAD and MIA (*p* = 0.0018 and 0.0013, Figure 3B). While LUAD had higher level of ctDNA fraction than MIA, no statistical significance was reached (*p* = 0.31). Similarly, early stages of lung cancer patients (stage I/II) contained significantly lower level of ctDNA than late stages (stage III/IV), with *p* value of 0.019.

### 3.4 Characteristics on BAL supernatant and precipitate

**FIGURE 4:**
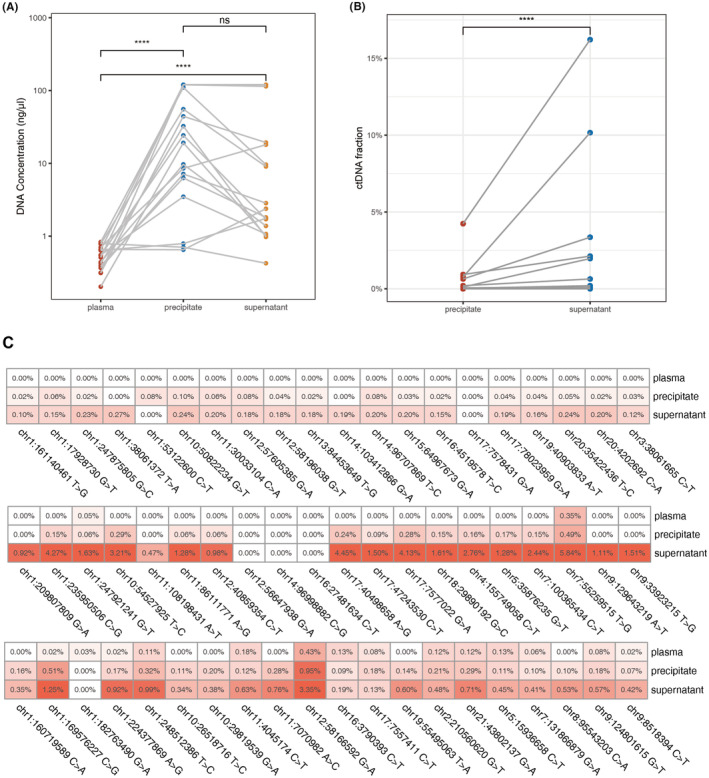
ctDNA analysis on plasma and BAL. (A) DNA concentrations in plasma, BAL supernatants, and BAL precipitates. (B) Comparison of ctDNA fractions between precipitates and supernatants in 8 BAL ctDNA‐positive patients. (C) Heatmap of 3 representative samples showing VAF on 20 designed loci in plasma, BAL supernatants, and precipitates. BAL, bronchoalveolar lavage; ctDNA, circulating tumor DNA.

We further assessed ctDNA detection rate in BAL supernatant and precipitate separately for 17 BAL samples. As illustrated in Figure 4A, both of BAL supernatant and BAL precipitate have significantly higher DNA concentration compared with plasma (Wilcoxon signed‐rank test, *p* < 0.001). Median DNA concentration was higher in precipitate (17.8 ng/μL) than in supernatant (6.62 ng/μL) but no significant difference was observed. No significant difference was observed in DNA concentration across disease stages (*p* = 0.11, 0.28) and histological types (*p* = 0.14, 0.07) in both supernatant and precipitates.

In 17 BAL samples, ctDNA was detected in 12 supernatants and 8 precipitates. All 8 ctDNA‐positive precipitates were have ctDNA detected in their corresponding supernatants. In the 8 ctDNA‐positive patients, we observed a significant upward trend of ctDNA fraction toward supernatants (Figure 4B, *p* < 0.001). Moreover, in the remaining 4 ctDNA‐positive supernatant‐only samples, ctDNA fractions were significantly lower compared to both ctDNA‐positive samples (Wilcoxon rank sum exact test, *p* = 0.004). As for individual variants identified in MarRyDa®, we also observed a significant correlation in allele frequencies among three types of samples. Figure 4C demonstrated VAF of each of 20 locus for three representative samples. In general, most of variants had lowest VAF in their plasma samples, while having highest VAF in corresponding BAL supernatant samples. Exceptions only occurred for the loci chr1:53122600C>T of the first sample and the loci chr1:182763490G>T of the third sample. Overall, supernatants showed consistency of high ctDNA fractions across all samples while plasma remained to the lowest level of ctDNA.

### 3.5 Impacts of sample quality control and panel design on ctDNA detection

**FIGURE 5:**
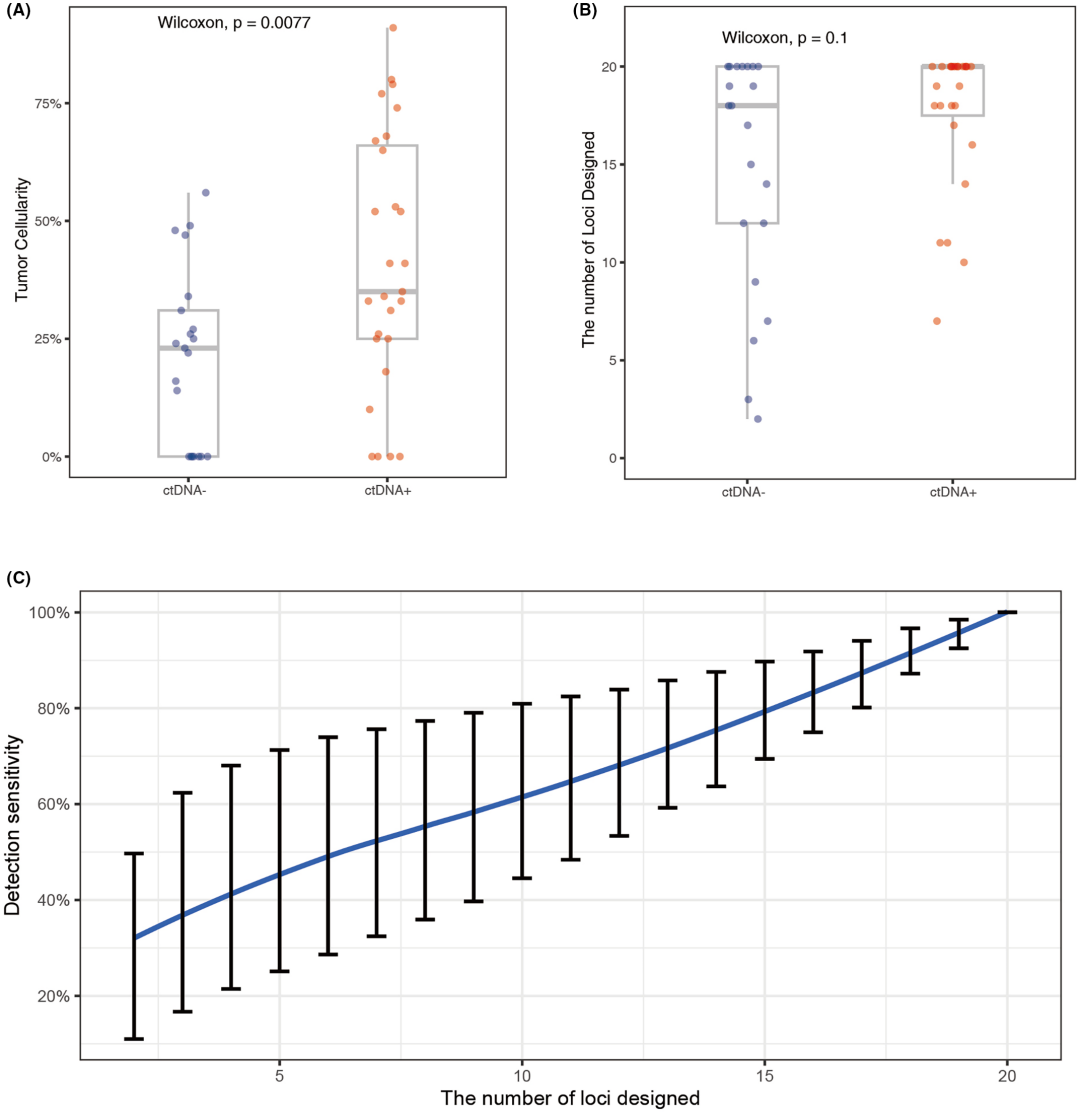
Evaluation of limiting factors for ctDNA detection sensitivity. (A) Boxplot of comparing tumor cellularity between ctDNA‐negative and ctDNA‐positive samples. (B) Boxplot of comparing the number of loci designed for each patient in ctDNA‐negative group and ctDNA‐positive group. (C) Detection sensitivity versus the number of loci designed for personalized ctDNA assay. ctDNA, circulating tumor DNA.

To explore the limits of quality control in ctDNA detection, we next sought to investigate the relationship between tumor cellularity and ctDNA positivity in plasma (Table S3). As shown in Figure 5A, ctDNA‐positive plasma had significantly higher cellularity than ctDNA‐negative plasma (*p* = 0.008). We hypothesized that the significance may cause by the limited number of loci being designed in low cellularity samples. Kendall's rank correlation analysis showed a slight but significant association between cellularity and mutation burden (*T* = 0.45, *p* < 0.0001), where the increase in tumor cellularity was associated with the increase in the number of mutations identified in WES. The association disappeared when we focused on samples with high levels of tumor cellularity (i.e., >50%, *T* = −0.04, *p* = 0.85). For tumor tissues with tumor cellularity less than 20%, only 31 variants were identified per sample, compared to 128 variants per sample for the high tumor cellularity group (≥20%). Furthermore, ctDNA‐negative plasma had lower median number of loci designed than ctDNA‐positive plasma but the difference was not significant (median: 18 vs. 20, *p* = 0.1).

To further investigate the impacts of the number of loci designed on ctDNA positivity, we performed random sampling on 15 ctDNA‐positive plasma samples containing 20 designed loci. Overall, as the number of sites included in the personalized panel dwindles, the sensitivity of detection decreases considerably compared with using all of 20 loci. With 10 loci being used for panel designing, detection sensitivity dropped to 61.86% (95% CI: 44.54%–80.93%). Sensitivity further dropped to 46.0% (95% CI: 25.11%–71.28%) when only 5 loci were used in panel designing.

## 4 DISCUSSION

Herein, we systematically investigated common drivers attributed to variability in ctDNA detection. We compared the level of ctDNA in supernatant and precipitate of BAL and showed that BAL supernatants are more abundant in tumor‐derived DNA than BAL precipitates. We also explored the potential of utilizing low‐tumor cellularity samples for personalized ctDNA assay designing. Our results suggested that ctDNA assay with a limited number of loci can still provide clinical values to patients; however, detection sensitivity is seriously deteriorated.

Although several previous studies showed the clinical utility of BAL fluid in genomic profiling,^17^, ^28^, ^29^ we characterized tumor‐specific somatic mutation in BAL via tumor tissue‐informed assay for the first time. Firstly, we demonstrated that DNA concentration were comparable in BAL supernatants and precipitates, and both of which were significantly higher than in plasma. The diminished DNA concentration in plasma signifies a reduced quantity of DNA being introduced into the sequencing library, which consequently results in scanty representation of DNA molecules that can be sequenced. Furthermore, we found BAL supernatants enriched in tumor‐derived DNA than both BAL precipitates and plasma. This finding is consistent with the previous study on lung cancer,^17^ supporting the idea of BAL supernatants being a better sequencing target than plasma. Nevertheless, enrichment of ctDNA in lavage fluid over plasma is not a universal trait across all cancer types, an example of which is endometrial cancer.^15^ We hypothesized that the variation between lavage supernatants and precipitates among different cancer types may arise from variation in field cancerization, a situation where histological normal adjacent cells around cancer origin showed genomic aberration. Since DNA molecules obtained from BAL supernatants are mostly cell‐free DNA while majority of DNA molecules in precipitates are genomic DNA from exfoliated cells. Cancers like endometrial cancer with high prevalence of field cancerization are more likely to have ctDNA detected in precipitated cell pellets.

Sample quality control is a crucial factor that can significantly impact the accuracy and reliability of clinical examination results. Setting high criteria for sample quality is essential to ensure accurate testing results; however, it also means a significant number of samples being rejected or discarded, leading to wastage and even additional costs (i.e., re‐sampling). We hereby showed that the number of loci used in personalized ctDNA detection assay is the one of major confounding factors for ctDNA detection sensitivity. Meanwhile, the number of loci designed is significantly limited by tumor tissue cellularity. In our cohort, 50% of samples with tumor cellularity less than 20% had less than 20 variants being detected in WES, which causes a considerable impact on panel design. Nevertheless, an assay with limited number of loci designed does provide some values. We argued that patients whose tissue samples failed quality control can still have their personalized ctDNA panels designed if access to alternative samples is not possible. A ctDNA‐negative result may not be fully trustworthy, but a ctDNA‐positive result could indicate the existence of cancer progression.

There are several limitations to our study. Firstly, most of our samples had early‐stage lung cancer, which impedes the statistical power of identifying differences across disease stages. Secondly, our research only investigated single nucleotide variants and small indel in BAL and plasma. Structural variations such as copy number variants and fusions were not analyzed in our study. Moreover, our study focused primarily on assay performance, delivering limited clinical benefits. Sequencing on a relatively large panel will potentially answer the question regarding structural variation.

Our study highlights the clinical significance of BAL supernatant in detecting tumor‐derived DNA. We showed that BAL supernatants carry more ctDNA than BAL precipitates, both of which are more informative than peripheral blood. However, BAL faces several challenges in clinical practice. One major challenge is that BAL is more difficult to retrieve than plasma, consequently, longitudinal surveillance based on BAL may be infeasible. Moreover, we demonstrated that limited but valuable information can be provided in low‐tumor cellularity samples. For patients who cannot retrieve a better tissue sample, performing a personalized ctDNA testing may still be worth a try. Overall, we elucidate that BAL supernatant may serve as a desirable target in cancer research. Also, setting a more flexible criteria for cellularity may still be beneficial in clinics.

## AUTHOR CONTRIBUTIONS

**Haihua Huang:** Conceptualization (equal); formal analysis (lead); validation (lead). **Zhentian Kai:** Formal analysis (equal); software (equal); visualization (equal); writing – original draft (lead). **Yuchen Wang:** Investigation (equal); resources (equal); writing – original draft (equal). **Xiaomiao Zhang:** Data curation (equal); methodology (equal). **Jin Wang:** Data curation (equal); methodology (equal). **Wei Zhang:** Investigation (equal); resources (equal). **Qian Xue:** Investigation (equal); resources (equal). **Hang Zhang:** Investigation (equal); resources (equal). **Hansong Jin:** Methodology (equal); resources (equal). **Peize Meng:** Methodology (equal); resources (equal). **Shuilong Zhang:** Software (equal); visualization (equal). **Yueyue Yang:** Methodology (equal). **Honghua Yang:** Methodology (equal). **Wanning Liang:** Validation (equal); writing – original draft (equal); writing – review and editing (equal). **Guangbing Zha:** Methodology (equal). **Peng Luo:** Supervision (equal); validation (equal). **Yan Xu:** Supervision (equal); validation (equal). **Weiwei Shi:** Supervision (equal); validation (equal). **Zheng Ruan:** Conceptualization (equal); project administration (lead); resources (equal); supervision (equal); validation (equal); writing – review and editing (equal).

## FUNDING INFORMATION

None.

## CONFLICT OF INTEREST STATEMENT

Z.K., S.Z, Y.Y, H.Y., W.L., G.Z., P.L., Y.X., and W.S are employees of Zhejiang Shaoxing Topgen Biomedical Technology Co., Ltd. All other authors have declared no conflicts of interest.

## ETHICS STATEMENT

Shanghai General Hospital Institutional Review Board has approved this study (No. 2023‐176). All participants had their written consent obtained.

## Supporting information


Figure S1.

Table S1.
Table S2.
Table S3.

## Data Availability

The data that support the findings of this study are available on request from the corresponding author. The data are not publicly available due to privacy or ethical restrictions.
