# Supplementary material for: Evaluating personalized circulating tumor DNA detection for early‐stage lung cancer
Source: Cancer Med. 2023 Dec 19;13(10):e6817. doi: 10.1002/cam4.6817 (PMC11112296; doi:10.1002/cam4.6817)
Supplement: Supplementary file 1 — Figure S1. [file CAM4-13-e6817-s001.docx]

Supplementary Results

Principle of designing of MarRyDa^®^ assay

The foundation of ctDNA detection lies in the ability to capture tumor-derived variants during sampling processes. To simulate the stochastic sampling variation, we applied Possion distribution to calculate the theoretical probability of detecting at least one tumor-derived mutant at the ctDNA concentration of 0.02%. By assuming conversion rate of cfDNA molecules to library is around 50%[1], we found that the theoretical probability of capturing at least one mutant initially increase rapidly with more mutants being traced. However, it soon reached its bottleneck at 20 mutants and only have marginal benefits if more mutants are included (Supplementary Figure S1). As the results, we designed the ctDNA detection panel MarRyDa^®^ that incorporated 20 tumor-derived loci, which could balance the sensitivity of ctDNA detection and the complexity of the assay development.


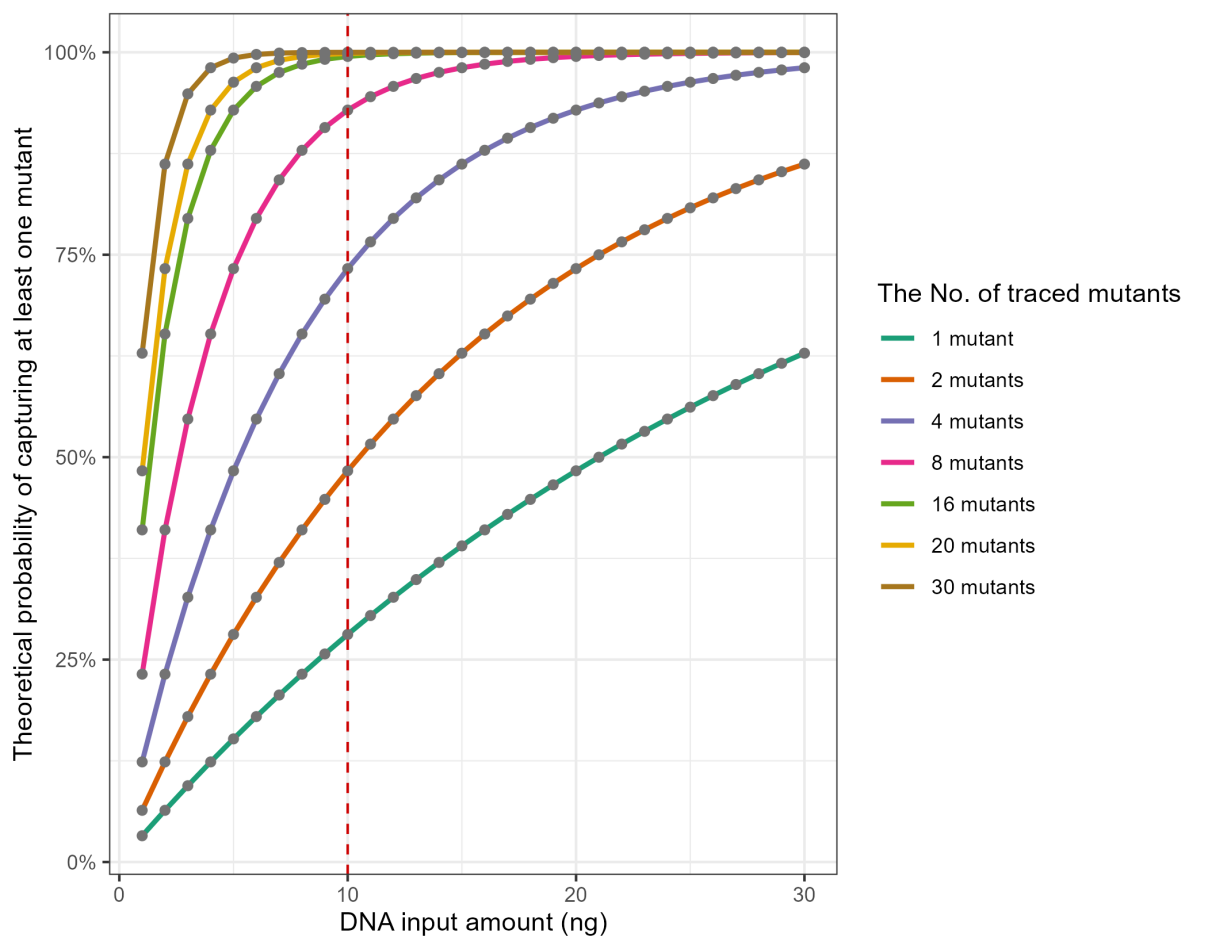


Supplementary Figure S1: Theoretical probability of capturing at least one mutant at varying DNA input amount and the number of traced mutants.

Reference:

1. Chin, Y.M. *et al.* (2020) ‘Ultradeep targeted sequencing of circulating tumor DNA in plasma of early and Advanced Breast Cancer’, *Cancer Science*, 112(1), pp. 454–464. doi:10.1111/cas.14697.
